# Verified Software Units

**DOI:** 10.1007/978-3-030-72019-3_5

**Published:** 2021-03-23

**Authors:** Lennart Beringer

**Affiliations:** Imperial College, London, UK; Princeton University, Princeton, NJ 08544 USA

**Keywords:** Verified Software Unit, Abstract Predicate Declaration, Residual Predicate, Positive Subtyping, Verified Software Toolchain

## Abstract

Modularity - the partitioning of software into units of functionality that interact with each other via interfaces - has been the mainstay of software development for half a century. In case of the C language, the main mechanism for modularity is the compilation unit / header file abstraction. This paper complements programmatic modularity for C with modularity idioms for specification and verification in the context of Verifiable C, an expressive separation logic for CompCert Clight. Technical innovations include (i) *abstract predicate declarations* – existential packages that combine Parkinson & Bierman’s abstract predicates with their client-visible reasoning principles; (ii) *residual* predicates, which help enforcing data abstraction in callback-rich code; and (iii) an application to pure (Smalltalk-style) objects that connects code verification to model-level reasoning about features such as subtyping, *self*, inheritance, and late binding. We introduce our techniques using concrete example modules that have all been verified using the Coq proof assistant and combine to fully linked verified programs using a novel, abstraction-respecting component composition rule for Verifiable C.

## 1 Introduction

Separation logic [61,53] constitutes a powerful framework for verifying functional correctness of imperative programs. Foundational implementations in interactive proof assistants such as Coq exploit the expressiveness of modern type theory to construct semantic models that feature higher-order impredicative quantification, step-indexing, and advanced notions of ghost state [4,36]. On the basis of proof rules that are justified w.r.t. the operational semantics of the programming language in question, these systems perform symbolic execution and employ multiple layers of tactical or computational proof automation to assist the engineer in the construction of concrete verification scripts. Perhaps most importantly, these implementations integrate software verification and model-level validation, by embedding assertions shallowly in the proof assistant's ambient logic; this permits specifications to refer to executable model programs or domain-specific constructions that are then amenable to code-independent analysis in Coq.

To realize the potential of separation logic, such implementations must be provided for mainstream languages and compatible with modern software engineering principles and programming styles. This paper addresses this challenge for Verifiable C, the program logic of the Verified Software Toolchain (VST [4]). We advance Verifiable C's methodology as follows.

1. We provide general infrastructure for *modular verification of modular programs* by extending Beringer and Appel's recent theory of function specification subsumption and intersection specifications [15] to a formal calculus for composing *verified software units* (VSUs) at their specification interface. Each VSU equips a compilation unit's header file with VST specifications of its API-exposed functions. Composition of VSUs matches the respective import and export interfaces, applying subsumption as necessary. Crucially, a compilation unit's private functions remain hidden and only need to be specified locally. Composition is compatible with source-level linking for CompCert Clight and supports repeated import of library modules (§3).
2. Utilizing existential abstraction [46] and parametricity, we extend work on abstract predicates [56] to provide clients with specification interfaces that differ in the degree to which module-internal representation details are revealed. This flexibility is achieved by codifying how the reasoning principles associated with a predicate can be selectively communicated to clients, using a device we call *(existentially) abstract predicate declarations* (APDs) (§4).
3. To investigate specification modularity in the presence of callbacks, we study variants of the subject-observer design pattern; we demonstrate that by complementing a module's *primary* predicate with *residual* predicates, representation hiding can be respected even at transient interaction points, where an invocation of a module's operation is interrupted, the module's invariant may be violated, and yet its internal state must remain unmodified and unexposed until the operation is resumed (§5).
4. We present a novel approach to foundational reasoning about object principles that modularly separates C code verification from model-level behavior. Exploiting the theory of positive subtyping [30], we cover subtyping, interfaces with multiple implementations, dynamic dispatch, *self*, and late binding, for a simple Smalltalk-style object model with static typing (§6).

This paper is accompanied by a development in Coq [14] that conservatively extends VST with the VSU infrastructure and contains several case studies. In addition to the examples detailed in the paper, the Coq code treats (i) the running example ("piles") of Beringer and Appel's development [15]; we retain their ability to substitute representation-altering but specification-preserving implementations; (ii) a variant of Barnett and Naumann's Master-Clock example [12], as another example of tightly coupled program units; and (iii) an implementation of the Composite design pattern, obtained by transcribing a development from the Verifast code base [35]. In addition, a VSU interface that unifies the APIs of $B^+$-trees and tries was recently developed by Kravchuk-Kirilyuk [40].

To see how APDs build on Parkinson and Bierman's work, consider a *concrete* representation predicate in the style of Reynolds [61]: $\mathrm{list}\ \mathrm{x}\ \alpha\ \mathrm{p}$ specifies that address $p$ represents a monotone list $\alpha$ of numbers greater than $x$:

$$\mathrm{list}\ \mathrm{x}\ \mathrm{nil}\ \mathrm{p} \stackrel{\mathrm{def}}{=} (\mathrm{p}{=}\mathsf{null})\ \&\ \mathsf{emp} \qquad \mathrm{list}\ \mathrm{x}\ (\mathrm{a}{::}\alpha)\ \mathrm{p} \stackrel{\mathrm{def}}{=} \exists\, \mathrm{q}.\ \mathrm{a} > \mathrm{x}\ \&\ \mathrm{p} \mapsto \mathrm{a}, \mathrm{q} * \mathrm{list}\ \mathrm{a}\ \alpha\ \mathrm{q}.$$

Being defined in terms of $\mapsto$, this definition assumes a specific data layout (a two-field **struct**). Representation-specific predicates enable verification of concrete implementations of operations such as *reverse*. But a client-facing specification of the entire list module should only expose the predicate in its folded form – a simple case of an abstract predicate. Indeed, while VST fully supports API exposure of **struct**s (incl. stack allocation), all examples in this paper employ an essentially "dataless" programming discipline [8,60,37] in which **struct**s are at most exposed as forward declarations. Clearly, such programmatic encapsulation should not be compromised through the use of concrete predicate definitions.

To regulate whether a predicate is available in its abstract or unfolded form at a particular program point, Parkinson and Bierman employ a notion of scope: predicates are available in their unfolded form when in scope and are treated symbolically elsewhere. This separation can naturally align with the partitioning into compilation units, but is all-or-nothing. But even in the absence of specifications, *different clients need different interfaces*: C developments routinely provide multiple header files for a single code unit, differing in the amount to which representational information is exposed. Mundane examples include special-purpose interfaces for internal performance monitoring or debugging. Extending this observation to specifications means supporting multiple public invariants. Indeed, several levels of visibility are already conceivable for our simple list predicate:

- no (un)folding, no exposed reasoning principles: properties that follow from the predicate's definition cannot be exploited during client-side verification;
- no (un)folding, but reasoning principles are selectively exposed; for example, one may expose the model-level property that $\alpha$ is strictly increasing, or the fact that the head pointer is null exactly if $\alpha$ is empty;
- the set of exposed reasoning principles includes fold/unfold lemmas (perhaps with the least-fixed-point property inherent in the inductive definition of the predicate), but the internal representation of nodes is encapsulated using a further predicate; hence, implementations are free to select a different **struct** layout, for example by swapping the order of fields;
- the predicate definition is fully exposed, including the internal data layout.

APDs support such flexibility by combining zero or more abstract predicate *declarations* (no definitions, to maintain implementation-independence) with axioms that selectively expose the predicates' reasoning principles. In parallel to programmatic forward declarations, an APD is exported in the specification interface of an API and is substantiated – in implementation-dependent fashion – in the VST proof of the corresponding compilation unit. This substantiation includes the validation of the exposed axioms. When specifying the API of a module, the engineer may not only refer to any APDs introduced by the module in question, but may also assume APDs for data structures provided by other modules (whose header files are typically **#include**d in the API in question). Matching the APD assumptions and provisions of different modules occurs naturally during the application of our component linking rule, ensuring that fully linked programs contain no unresolved APD assumptions.

Before going into technical details, we first summarize key aspects of VST.

## 2 Program verification using VST

Verification using VST happens exclusively inside the Coq proof environment, and operates directly on abstract syntax trees of CompCert Clight. Typically, these ASTs result from feeding a C source file through CompCert's frontend, clightgen, but they may also originate from code synthesis. Either way, verification applies to the same code that is then manipulated by CompCert's optimization and backend phases. This eliminates the assurance gap that emerges when a compiler's (intermediate) representation diverges syntactically or semantically from a verification tool's representation. The absence of such gaps is the gist of VST's machine-checked soundness proof: verified programs are safe w.r.t. the operational semantics of Clight; this guarantee includes memory safety (absence of null-pointer dereferences, out-of-bounds array accesses, use-after-frees,. . . ) but also absence of unintended numeric overflows or race conditions. As Clight code is still legal C code (although slightly simplified, and with evaluation order determinized), verification happens at a level the programmer can easily grasp.

In contrast to other verification tools, VST does not require source code to be annotated with specifications. Instead, the verification engineer writes specifications in a separate Coq file. By not mixing specifications (let alone aspects of proof, such as loop invariants) with source code, VST easily supports associating multiple specifications with a function and constructing multiple proofs for a given code/specification pair.

We write function specifications $\phi$ in the form $\{P\} \leadsto \{v.\ Q\}$ where $v$ denotes the (sometimes existentially quantified) return value and $P$ and $Q$ are separation logic assertions. To shield details of its semantic model, VST exposes heap assertions using the type **mpred** rather than as direct Coq-level predicates. On top of **mpred**, assertions are essentially embedded shallowly, giving the user access to the logical and programmatic features of Coq when defining specifications.

VST's top-level notion asserting that a (closed) program $p$ – which must include main, with a standard specification – has been verified in Coq is $\vdash p : G$ ("**semax_prog**"). Here, $G$ – of type **funspecs**, i.e. associating specifications $\phi$ to function identifiers $f$ – constitutes a witnessing proof context that contains specifications for all functions in $p$ and must itself be justified: for each $(f, \phi_f) \in G$, the user must exhibit a Coq proof of $G \vdash f : \phi_f$ ("**semax_body**"), expressing that $f$ satisfies $\phi_f$ under hypotheses in $G$. VST's step-indexed model ensures logical consistency in case of (mutual) recursion.

We exploit Beringer and Appel [15]'s theory of specification *subsumption* $\phi <: \psi$ which extends parameter adaptation [38,50,48] to step-indexed separation logics for C and allows a function verified w.r.t $\phi$ to be used by clients expecting specification $\psi$. This theory includes a notion of specification *intersection* $\wedge$ which – similar to, e.g. the `also` combinator of the Java Modelling Language (JML, [19])– allows functions to have multiple specifications. Noticeably, subsumption and intersection are related in formally the same manner as intersection types and subtyping are in type theory: in particular, they satisfy the laws $\phi_1 \wedge \phi_2 <: \phi_i$ (for $i \in \{1, 2\}$) and $\dfrac{\psi <: \phi_1 \quad \psi <: \phi_2}{\psi <: \phi_1 \wedge \phi_2}$ (cf. [58], page 206).

## 3 VSU calculus

As described above, VST verification amounts to exhibiting a $G$ with $\vdash p : G$. In contrast to VST's previous linking regime, VSU ensures existence of $G$ during component linking without actually constructing $G$, maintaining representation hiding and non-exposure of private functions. Indeed, the modules' specification interfaces (specs of imported and exported functions) suffice for proving that a suitable $G$ exists, as long as each module's individual justification includes the verification of its private functions.

### 3.1 Components and soundness

VSU extends CompCert's distinction between internal functions (those equipped locally with a function body) and external functions (functions defined in other compilation units, incl. system functions). Given a Clight compilation unit $p$, we denote these (disjoint) sets by $\mathit{IntFuns}(p)$ and $\mathit{ExtFuns}(p)$, respectively. VSU further distinguishes between system functions (typically provided by the OS) and ordinary external functions: the former ones are not expected to be verified using VST even in a fully linked program, so VSU merely records their use.

VSU's main judgment is $\vdash^{\mathcal{S}}_{P} [\mathcal{I}]\, p\, [\mathcal{E}]$, to be read as *using specified imports* $\mathcal{I}$ *and system functions* $\mathcal{S}$, *p provides/ exports functions (with specifications)* $\mathcal{E}$, *using internal memory satisfying (initially)* $P$. The entities $\mathcal{S}$, $\mathcal{I}$, and $\mathcal{E}$ are all **funspecs**, while $P$ specifies the memory holding $p$'s global variables; $P$'s formal type is **globals** $\rightarrow$ **mpred** where **globals** refers to a map from global identifiers to CompCert values.

The judgment $\vdash^{\mathcal{S}}_{P} [\mathcal{I}]\, p\, [\mathcal{E}]$ is formally introduced as an existential abstraction (in Coq: a **Record** type) over a proof context $G$, which is again of type **funspecs**:

$$\vdash^{\mathcal{S}}_{P} [\mathcal{I}]\, p\, [\mathcal{E}] \stackrel{\text{def}}{=} \exists G.\ G \vdash^{\mathcal{S}}_{P} [\mathcal{I}]\, p\, [\mathcal{E}].$$

The role of $G$ is to serve as the witness justifying the specification interface; as such it associates specifications also to $p$'s private functions; existentially hiding it shields implementation details.

The formation of the lower-level judgment $G \vdash^{\mathcal{S}}_{P} [\mathcal{I}]\, p\, [\mathcal{E}]$ is subject to the following constraints:

**Definition 1.** *Proof context G justifies a component (specification) for Clight compilation unit p with respect to system calls* $\mathcal{S}$*, imports* $\mathcal{I}$*, exports* $\mathcal{E}$*, and predicate P, notation* $G \vdash^{\mathcal{S}}_{P} [\mathcal{I}]\, p\, [\mathcal{E}]$*, if*

1. $\mathit{dom}\,\mathcal{I} \cap \mathit{dom}\,\mathcal{S} = \emptyset$ *and* $\mathit{dom}\,\mathcal{I} \cup \mathit{dom}\,\mathcal{S} \subseteq \mathit{ExtFuns}(p)$,
2. $\mathit{dom}\,G = \mathit{IntFuns}(p) \cup \mathit{dom}\,\mathcal{S}$, *with* $G(i) = \mathcal{S}(i)$ *whenever* $i \in \mathit{dom}\,\mathcal{S}$,
3. $\mathit{dom}\,\mathcal{E} \subseteq \mathit{dom}\,G$, *with* $G(i) <: \mathcal{E}(i)$ *for all* $i \in \mathit{dom}\,\mathcal{E}$,
4. $\mathcal{I} \cup G \vdash_{\mathsf{func}} \mathit{funs}_p : G$,
5. $\forall g, \mathsf{InitGPred}(\mathsf{Vardefs}(p))\ g \vdash P\, g$

The first three clauses are largely administrative; they express, respectively, that (1) system functions and imported functions are disjoint sets of external functions, (2) $G$ contains specifications for exactly the system functions and the internal functions, and (3) all exported specifications are abstractions of entries in $G$, in the sense of specification subsumption $<:$.

Clause (4) constitutes the main proof obligation and refers to a slight refactoring of VST's function-verification judgment $G_1 \vdash_{\mathsf{func}} \mathit{funs} : G_2$ (**semax-func**), where *funs* associates CompCert function definitions with identifiers. The instantiation $\mathcal{I} \cup G \vdash_{\mathsf{func}} \mathit{funs}_p : G$ hence requires that imports $\mathcal{I}$ suffice for justifying all entries in $G$: each system function specification in $G$ must be valid, and each specification of an internal function must be justified by a VST proof the corresponding function body in *funs*; calls to internal and system functions inside the body are resolved by reference to $G$, and calls to external functions are resolved by the import specifications, $\mathcal{I}$.

Finally, clause (5) requires $p$'s global variables to collectively satisfy $P$ (after initialization) but avoids referring to these variables by name.

We point out two further aspects of Definition 1. First, we note that system functions may be exported (we do not require $\mathit{dom}\,\mathcal{S} \cap \mathit{dom}\,\mathcal{E} = \emptyset$), and that imports and exports are distinct ($\mathit{dom}\,\mathcal{I} \cap \mathit{dom}\,\mathcal{E} = \emptyset$ follows). Second, we note that for $\mathcal{I} = \emptyset$, clause (4) yields $G \vdash_{\mathsf{func}} \mathit{funs}_p : G$, i.e. the heart of VST's soundness condition **semax_prog** for programs comprised of a single compilation unit. Hence, the goal of VSU verification is to exhaustively apply VSU's combination rule (presented in the next subsection) until all imports have been resolved.

Once a component has been verified and is exposed as $\vdash^{\mathcal{S}}_{P} [\mathcal{I}]\, p\, [\mathcal{E}]$, the specifications of $p$'s private functions are hidden inside the existentially quantified context $G$ and hence inaccessible.

### 3.2 Derived rules

It is easy to derive a rule of consequence from Definition 1 that strengthens imports and relaxes exports:

$$\frac{\mathcal{I}' <: \mathcal{I} \qquad \vdash^{\mathcal{S}}_{P} [\mathcal{I}]\, p\, [\mathcal{E}] \qquad \mathcal{E} \sqsubseteq \mathcal{E}' \qquad \forall\, g, P\, g \vdash P'\, g}{\vdash^{\mathcal{S}}_{P'} [\mathcal{I}']\, p\, [\mathcal{E}']}\,\textsc{VSUConseq}$$

For imported functions, we require pointwise subsumption, by defining $\mathcal{I}' <: \mathcal{I}$ to hold if $\mathit{dom}\,\mathcal{I} = \mathit{dom}\,\mathcal{I}'$ and $\mathcal{I}'(i) <: \mathcal{I}(i)$ for all $i \in \mathit{dom}\,\mathcal{I}$. On the export side, we allow hiding of entries, by defining $\mathcal{E} \sqsubseteq \mathcal{E}'$ to hold if $\mathit{dom}\,\mathcal{E}' \subseteq \mathit{dom}\,\mathcal{E}$ and $\mathcal{E}(i) <: \mathcal{E}'(i)$ for all $i \in \mathit{dom}\,\mathcal{E}'$. The calculus is invariant in the specifications of system functions, but allows weakening of the initialization predicate. The derivation of this rule instantiates the context witnessing the concluding judgment by the (abstract) witness obtained from unfolding the hypothetical judgment.

VSU's workhorse is the composition rule, VSULink, shown in Figure 1. The side conditions treat the components symmetrically and are motivated as follows. The rule constructs a component specification for a linked program $p$ that retains the internal functions of $p_1$ and $p_2$, and also any unresolved external functions, as

$$
\begin{array}{lc}
(a) & \vdash^{\mathcal{S}_1}_{P_1} [\mathcal{I}_1]\, p_1\, [\mathcal{E}_1] \qquad \vdash^{\mathcal{S}_2}_{P_2} [\mathcal{I}_2]\, p_2\, [\mathcal{E}_2] \\[1em]
(b) & \begin{array}{c} \forall i \in \mathit{IntFuns}(p_1) \cup (\mathit{ExtFuns}(p_1) \setminus \mathit{IntFuns}(p_2)), p(i) = p_1(i) \\ \forall i \in \mathit{IntFuns}(p_2) \cup (\mathit{ExtFuns}(p_2) \setminus \mathit{IntFuns}(p_1)), p(i) = p_2(i) \\ \mathit{dom}\, p = \mathit{dom}\, p_1 \cup \mathit{dom}\, p_2 \end{array} \\[1em]
(c) & \begin{array}{c} \forall i \in (\mathit{IntFuns}(p_1) \cap \mathit{IntFuns}(p_2)) \cup (\mathit{ExtFuns}(p_1) \cap \mathit{ExtFuns}(p_2)),\ p_1(i) = p_2(i) \\ \forall i \in \mathit{IntFuns}(p_1) \cap \mathit{ExtFuns}(p_2),\ \mathit{sig}(p_1(i)) = \mathit{sig}(p_2(i)) \wedge i \in \mathit{dom}\, \mathcal{I}_2 \\ \forall i \in \mathit{IntFuns}(p_2) \cap \mathit{ExtFuns}(p_1),\ \mathit{sig}(p_2(i)) = \mathit{sig}(p_1(i)) \wedge i \in \mathit{dom}\, \mathcal{I}_1 \end{array} \\[1em]
(d) & \mathit{dom}\, \mathcal{S}_1 \cap \mathit{IntFuns}(p_2) = \emptyset \qquad \mathit{dom}\, \mathcal{S}_2 \cap \mathit{IntFuns}(p_1) = \emptyset \\[1em]
(e) & \begin{array}{c} \forall i \in \mathit{dom}\, \mathcal{I}_2 \cap (\mathit{dom}\, \mathcal{S}_1 \cup \mathit{IntFuns}(p_1)),\ i \in \mathit{dom}\, \mathcal{E}_1 \wedge \mathcal{E}_1(i) <: \mathcal{I}_2(i) \\ \forall i \in \mathit{dom}\, \mathcal{I}_1 \cap (\mathit{dom}\, \mathcal{S}_2 \cup \mathit{IntFuns}(p_2)),\ i \in \mathit{dom}\, \mathcal{E}_2 \wedge \mathcal{E}_2(i) <: \mathcal{I}_1(i) \end{array} \\[1em]
(f) & \forall i \in \mathit{dom}\, \mathcal{I}_1 \cap \mathit{dom}\, \mathcal{I}_2,\ \mathcal{I}_1(i) = \mathcal{I}_2(i) \\[1em]
(g) & \mathcal{I} = \mathcal{I}_1 \setminus (\mathit{dom}\, \mathcal{S}_2 \cup \mathit{IntFuns}(p_2)) \cup \mathcal{I}_2 \setminus (\mathit{dom}\, \mathcal{S}_1 \cup \mathit{IntFuns}(p_1)) \\[1em]
(h) & \mathsf{Vardefs}(p_1) \cap \mathsf{Vardefs}(p_2) = \emptyset \quad \mathsf{Vardefs}(p_1) \cup \mathsf{Vardefs}(p_2) = \mathsf{Vardefs}(p) \\
\hline
 & \vdash^{\mathcal{S}_1 \mathbin{/\!\!\wedge\!\!\backslash} \mathcal{S}_2}_{P_1 * P_2} [\mathcal{I}]\, p\, [\mathcal{E}_1 \mathbin{/\!\!\wedge\!\!\backslash} \mathcal{E}_2]
\end{array}
$$

**Fig. 1.** VSU's rule of component composition, VSULink.

detailed in side conditions $(b)$. Condition $(c)$ requires functions classified identically by $p_1$ and $p_2$ to have identical definitions, and requires differently classified functions to have identical type signatures and be in the import set of the compilation unit not providing the implementation. Condition $(d)$ formalizes that system functions are not locally defined in either unit. Condition $(e)$ expresses that a function imported by one module and programmatically provided by the other module must be exported by the provider; this condition ensures that the export contract cannot be bypassed. Condition $(f)$ expresses that functions imported by both units must be imported identically - if necessary, this can be achieved using the consequence rule. Condition $(g)$ calculates the remaining import specifications by combining the constituent imports, removing entries for the resolved functions, and ensuring the absence of duplicates. The final condition, $(h)$, mandates that global variables from $p_1$ and $p_2$ be distinct (hence initialization predicates have disjoint footprints) and propagated to $p$.

The most interesting aspect of the rule is the duplicate use of the intersection operator, $C_1 \mathbin{/\!\!\wedge\!\!\backslash} C_2$, for constructing the concluding specifications of exported functions and system functions. The general definition of this operator is

$$
C_1 \mathbin{/\!\!\wedge\!\!\backslash} C_2 := \lambda i. \begin{cases} C_1(i) \wedge C_2(i) & \text{if } i \in \mathit{dom}\, C_1 \cap \mathit{dom}\, C_2 \\ C_1(i) & \text{if } i \in \mathit{dom}\, C_1 \setminus \mathit{dom}\, C_2 \\ C_2(i) & \text{if } i \in \mathit{dom}\, C_2 \setminus \mathit{dom}\, C_1 \end{cases}
$$

where $\wedge$ denotes the specification intersection operator mentioned in Section 2. Thus, exporting $\mathcal{E}_1 \mathbin{/\!\!\wedge\!\!\backslash} \mathcal{E}_2$ effectively exports both $\mathcal{E}_1$ and $\mathcal{E}_2$, and similarly for $\mathcal{S}_1 \mathbin{/\!\!\wedge\!\!\backslash} \mathcal{S}_2$. Indeed, the individual export specifications can be reestablished using the consequence rule, as the properties of intersection specifications mentioned in Section 2 lift to (export specification) contexts: we have $C_1 \mathbin{/\!\!\wedge\!\!\backslash} C_2 \sqsubseteq C_i$ for $i \in \{1, 2\}$ and $\dfrac{X \sqsubseteq C_1 \quad X \sqsubseteq C_2}{X \sqsubseteq C_1 \mathbin{/\!\!\wedge\!\!\backslash} C_2}$ for any $X$.

By permitting functions $f$ that are internal to both $p_1$ and $p_2$, VSU supports diamond-shaped composition patterns in which a sub-component, e.g. a library, is imported multiple times. Conditions ($b$) and ($c$) ensure that all copies of a repeatedly imported function $f$ have the same body (i.e. CompCert AST), and that this body is retained in $p$. However, the library's export specification may have been imported differently by the different units, hence $G_1$ and $G_2$ may well associate different (and formally incompatible) specifications with $f$. As $G_1$ and $G_2$ are existentially hidden, we cannot inspect these specifications: adding a side condition to the rule that mentions the specifications $G_1(f)$ and $G_2(f)$ would violate the abstraction principle. Nevertheless, the proof of the composition rule still requires us to attach *some* specification to the shared function, when constructing the witnessing context of the concluding judgment, $G$. Our solution is to use intersection $\mathbin{/\!\!\wedge\!\!\backslash}$, i.e. to instantiate the witness $G$ with $G_1 \mathbin{/\!\!\wedge\!\!\backslash} G_2$ in the Coq proof of VSULink. By terminating the Coq proof script with **Qed** rather than **Defined**, this instantiation is opaque to clients: applications of VSULink during program verification merely see that *some* $G$ exists.

Most side conditions of the rule are computational; in our applications of the rule in Sections 4.5 and 5, Coq's tactical engine solves the majority of them.

## 4 APDs and specification interfaces

We now turn to the organization of predicates and function specifications. Our organization reflects typical realizations of abstraction principles in C, where heap data structures are introduced using forward declarations and referred to via pointers in header files, while the selection of a concrete representation (perhaps using private static variables) is private to an implementation. We illustrate our approach using Parkinson and Bierman's connection pool example [56],

```
typedef struct database *Database;
typedef struct connection *Connection;
Connection consConn (Database d);
Database newDB (int DBidentifier);
```

```
#include "Connection.h"
typedef struct pool *Pool;
Pool consPool (Database d);
Connection getConn (Pool p);
void freeConn (Pool p, Connection c);
```

**Fig. 2.** Connection pools in C: Connection.h (left) and Connectionpool.h (right).

ported to C as an implementation of the APIs in Figure 2. Using forward declarations, the header files reveal only minimal information about the implementation. Connection.h allows clients to create a database entity (the parameter denotes a unique identifier; Parkinson and Bierman omit this constructor and do not model the type database explicitly) and to create connections to a database using the constructor consConn. Connectionpool.h models a collection of (dormant) connections associated with a database; clients construct a pool using consPool, request connections using getConn, and return them using freeConn.

### 4.1 Abstract predicate declarations (APDs)

Figure 3 introduces abstract predicate declarations for the three data structures. Each APD declares zero or more spatial predicates, i.e. **mpred**s relating a CompCert (pointer) value to suitable semantic information. Semantic information for the database is a DBindex (effectively a mathematical integer); connection and pool structures maintain pointers to the database; connections have additional internal state represented by the (abstract) type ConnTP.

**Record** DatabaseAPD := {
  DB: DBIndex → val → mpred;
  DB_ptrnull: ∀ db s, DB db s ⊢
    !!(is_pointer_or_null s) }.
**Record** PoolAPD := {
  CPool: val → val → mpred;
  CPool_ptrnull: ∀ d p, CPool d p ⊢
    !!(is_pointer_or_null p) }.

**Record** ConnectionAPD := {
  ConnTP:Type;
  Conn: (val ∗ ConnTP) → val → mpred;
  NextConn: ConnTP → globals → mpred;
  Conn_isptr: ∀ C c, Conn C c ⊢ !!(isptr c);
  Conn_validptr: ∀ C c, Conn C c ⊢
    valid_pointer c }.

**Fig. 3.** APDs for the connection pool example. val is CompCert's type of values.

Specifically, DatabaseAPD corresponds to the Database type declaration in Connection.h and asserts existence of a predicate DB, together with an axiom that enables clients to store a reference to a database in their own data structure. Operator !! injects a Coq proposition into VST's assertion language.

In similar style, ConnectionAPD and PoolAPD declare predicates Conn and CPool for the **struct** declarations connection and pool. In contrast to Parkinson and Bierman, we model that the connection module maintains state using the predicate NextConn. There is no need to reveal the concrete static variable used by our implementation though: **globals** denotes the collection of all such variables in VST. We assert that the head values of Conn and CPool are provably nonnull pointers and that a Conn's head pointer is furthermore valid.

All APDs are introduced as (dependent) **Record** types in Coq. We will construct values of these types in Section 4.3, i.e. implementation-dependent concrete predicate definitions and lemmas validating the axioms. But first, we use the APD types abstractly to introduce specifications for the two modules.

### 4.2 Abstract specification interfaces (ASIs)

*Abstract specification interfaces (ASIs)* consist of VST specifications for the API-exposed functions, parametric in all relevant APDs. In addition to the APDs introduced above, our example uses a third APD, denoted $\mathsf{M}$, that declares an abstract predicate $\mathsf{Mem}_\mathsf{M}\ \mathsf{gv}$ and represents the malloc/free library.

Figure 4 shows the ASI of Connection.h. We use subscripts to refer to the APD parameters: for example, $\mathsf{DB}_\mathsf{D}\ i\ p$ is the **mpred** obtained by applying the DB component of a database APD D to index $i$ and pointer value $p$.

| Function | Spec |
|---|---|
| $\mathsf{newDB}(i;\mathsf{gv})$ | $\{\mathsf{Mem}_\mathsf{M}\ \mathsf{gv}\} \leadsto \{p.\ \mathsf{DB}_\mathsf{D}\ i\ p * \mathsf{Mem}_\mathsf{M}\ \mathsf{gv}\}$ |
| $\mathsf{consConn}(d;\mathsf{gv})$ | $\{\mathsf{DB}_\mathsf{D}\ i\ d * \mathsf{NextConn}_\mathsf{C}\ c\ \mathsf{gv} * \mathsf{Mem}_\mathsf{M}\ \mathsf{gv}\} \leadsto$ $\left\{p.\begin{array}{l}\text{if } p = \mathsf{null} \text{ then } \mathsf{NextConn}_\mathsf{C}\ c\ \mathsf{gv} * \mathsf{DB}_\mathsf{D}\ i\ d * \mathsf{Mem}_\mathsf{M}\ \mathsf{gv} \\ \text{else } \exists c'.\ \mathsf{NextConn}_\mathsf{C}\ c'\ \mathsf{gv} * \mathsf{Conn}_\mathsf{C}\ (d,c)\ p * \mathsf{DB}_\mathsf{D}\ i\ d * \mathsf{Mem}_\mathsf{M}\ \mathsf{gv}\end{array}\right\}$ |

**Fig. 4.** ASI for Connection.h, parametric in databases (D), connections (C), and memory systems (M). $\mathsf{Mem}_\mathsf{M}\ \mathsf{gv}$ represents M's abstract predicate for a memory manager that is accessed by malloc and free.

A specification $\mathrm{F}(\vec{x};\mathsf{gv}) : \{Pre\} \leadsto \{v.\ Post\}$ is to be understood in safety-guaranteeing partial-correctness style, where $\vec{x}$ denotes a list of actual arguments (of type val), gv refers to (if present) the global environment, $v$ (again of type val) represents the return value (if present), and other items are implicitly universally quantified. Callers of such a function select instantiations for the universally quantified entities ("witnesses") and must then establish *Pre*.

Thus, the specification of newDB asserts that a new database entity satisfying $\mathsf{DB}_\mathsf{D}\ i\ p$ is allocated at the return value $p$, for the database with index $i$ (an input argument). The allocation draws upon the abstract predicate $\mathsf{Mem}_\mathsf{M}\ \mathsf{gv}$ which is "located" at some global variable that is private to the malloc/free library.

The specification of constructor consConn refers to $\mathsf{Mem}_\mathsf{M}\ \mathsf{gv}$ in similar fashion and advances the module's connection counter from $c$ to some $c'$ upon success; in contrast to Parkinson and Bierman, we also support unsuccessful requests.

The ASI for Connectionpool.h in Figure 5 is additionally parametric in an PoolAPD, P. Our specifications are again slightly more precise than the ones given by Parkinson and Bierman. As a consequence, the precondition of a sequence such as $p := \texttt{consPool}(s); c := \texttt{getConn}(s); \texttt{freeConn}(p, c)$ is $\mathsf{DB}_\mathsf{D}\ i\ d * \mathsf{Mem}_\mathsf{M}\ \mathsf{gv} * \mathsf{NextConn}_\mathsf{C}\ s\ \mathsf{gv}$ rather then emp, hence exposing the reliance on the memory manager etc..Prefixing the instruction $d := \texttt{newDB}(i)$ establishes $\mathsf{DB}_\mathsf{D}\ i\ d$; we will explain how the latter two conjuncts are provided in Section 4.5.

### 4.3 Verification of ASI-specified compilation units

Substantiating the ASI of a header file, means to give – for a concrete implementation – concrete definitions for the predicates in the newly introduced APDs,

| Function | Spec |
|---|---|
| $\mathsf{consPool}(d;\mathsf{gv})$ | $\{\mathsf{Mem}_\mathsf{M}\ \mathsf{gv}\} \leadsto \{p.\ \mathsf{CPool}_\mathsf{P}\ d\ p * \mathsf{Mem}_\mathsf{M}\ \mathsf{gv}\}$ |
| $\mathsf{getConn}(p;\mathsf{gv})$ | $\{\mathsf{CPool}_\mathsf{P}\ d\ p * \mathsf{DB}_\mathsf{D}\ i\ d * \mathsf{NextConn}_\mathsf{C}\ c\ \mathsf{gv} * \mathsf{Mem}_\mathsf{M}\ \mathsf{gv}\}$ $\leadsto \left\{ n. \begin{array}{l} \mathsf{CPool}_\mathsf{P}\ d\ p * \mathsf{DB}_\mathsf{D}\ i\ d * \mathsf{Mem}_\mathsf{M}\ \mathsf{gv} * \\ \mathsf{if}\ n = \mathsf{null}\ \mathsf{then}\ \mathsf{NextConn}_\mathsf{C}\ c\ \mathsf{gv} \\ \mathsf{else}\ \exists c'\ c''.\ \mathsf{NextConn}_\mathsf{C}\ c'\ \mathsf{gv} * \mathsf{Conn}_\mathsf{C}\ (d, c'')\ n \end{array} \right\}$ |
| $\mathsf{freeConn}(p,i;\mathsf{gv})$ | $\{\mathsf{CPool}_\mathsf{P}\ d\ p * \mathsf{Conn}_\mathsf{C}\ (d,c)\ i * \mathsf{Mem}_\mathsf{M}\ \mathsf{gv}\} \leadsto \{\mathsf{CPool}_\mathsf{P}\ d\ p * \mathsf{Mem}_\mathsf{M}\ \mathsf{gv}\}$ |

**Fig. 5.** The ASI for Connectionpool.h is parametric in a database APD (D), a connection APD (C), a connection pool APD (P), and a memory manager APD (M). As consPool takes a formal parameter $d$, the reader may have expected the specification $\{\mathsf{DB}_\mathsf{D}\ i\ d * \mathsf{Mem}_\mathsf{M}\ \mathsf{gv}\} \leadsto \{p.\ \mathsf{CPool}_\mathsf{P}\ d\ p * \mathsf{DB}_\mathsf{D}\ i\ d * \mathsf{Mem}_\mathsf{M}\ \mathsf{gv}\}$ which is indeed derivable from the one given using VST's frame rule.

show that these definitions validate the associated axioms, and finally construct a VSU that has the ASI's specifications as the export interface $\mathcal{E}$. All these constructions are parametric in the APDs provided by other modules.

We refer the reader to our source code [14] for the C implementation, the (concrete) predicate definitions, and the proofs of the APD-supporting axioms. In case of Connection.c, these proofs reveal the instantiation of the APD's ConnTP to Coq's type of integers, Z, corresponding to the existence of a global integer variable in the C code that maintains a connection counter; the corresponding $.\ \dot{\mapsto}_{.}\ .$ predicate then furnishes the abstract predicate NextConn.

The substantiations of a unit's APDs are subsequently used to instantiate its ASI and the specifications of its imported function, yielding (together with specifications of private functions) a proof context $G$ that the unit's local function bodies are then verified against. APDs provided by other compilation units are left abstract, so expose only their axioms. Specifically, the substantiation for Connection.c yields values $c$ and $d$ of types ConnectionAPD and DatabaseAPD, respectively, the predicate $N = \mathsf{NextConn}\ c\ 0$, and a VSU

$$\mathsf{VSU}_{\mathtt{Conn}} \stackrel{\text{def}}{=} \vdash^{\emptyset}_{N} [\mathcal{I}_{\mathtt{Conn}}]\ \mathit{Connection.prog}\ [\mathcal{E}_{\mathtt{Conn}}]$$

where $\mathcal{E}_{\mathtt{Conn}}$ is the partial specialization of the specifications in Figure 4 to C $= c$ and D $= d$, *Connection.prog* is CompCert's AST for Connection.c, and $\mathcal{I}_{\mathtt{Conn}}$ contains a specification for surelymalloc. For ConnectionPool.c, we similarly obtain a value $p$ of type ConnectionpoolAPD and a VSU

$$\mathsf{VSU}_{\mathtt{Pool}} \stackrel{\text{def}}{=} \vdash^{\emptyset}_{\mathsf{emp}} [\mathcal{I}_{\mathtt{Pool}}]\ \mathit{Connectionpool.prog}\ [\mathcal{E}_{\mathtt{Pool}}],$$

where $\mathcal{I}_{\mathtt{Pool}}$ is comprised of the (abstract) specification of consConn and specifications for free and surelymalloc, and $\mathcal{E}_{\mathtt{Pool}}$ is the partial specialization of Figure 5 to P $= p$. Both VSUs are parametric in M, but $\mathsf{VSU}_{\mathtt{Pool}}$'s additional parameters D and C are instantiated when $\mathsf{VSU}_{\mathtt{Conn}}$ and $\mathsf{VSU}_{\mathtt{Pool}}$ are combined using rule VSULink. The result, $\mathsf{VSU}_{\mathtt{CP}}$, is still parametric in M but has resolved the imports of consConn, leaving only imports for free and surelymalloc.

### 4.4 A VSU for a malloc-free library

A recent application of VST is Appel and Naumann's verification of a malloc/free library [5]. Internally maintaining a fixed number of freelists – for entities of different size – this library exposes four functions in its API: malloc, free, pre_fill, try_pre_fill. When porting this development to the VSU framework, these give rise to two ASIs. The first one contains specifications for all four functions and is suitable for resource-aware clients. It employs the APD MallocFree_R_APD:

**Record** MallocTokenAPD := {
  malloc_token': share → Z → val → mpred;
  malloc_token'_valid_pointer: ∀ sh sz p, malloc_token' sh sz p ⊢ valid_pointer p;
  malloc_token'_facts: ∀ sh sz p, malloc_token' sh sz p ⊢!! malloc_compatible sz p }.
**Record** MallocFree_R_APD :=
  { MF_Tok_R :> MallocTokenAPD; mem_mgr_R: resvec → globals → mpred }.

mem_mgr_R models the freelists as a resource vector that indicates the length of each freelist. The predicate malloc token' refers to the piece of memory that is typically located at a small negative offset of a malloc'ed entity and holds administrative information of the library, but conceptually, it also constitutes a token that enables clients to share malloc'ed entities among different threads without loosing the ability to safely free entities. The second ASI only exposes malloc and free, and employs the more abstract APD

**Record** MallocFreeAPD :=
  { MF_Tok :> MallocTokenAPD; mem_mgr: globals → mpred }.

MF_Tok still presents a malloc token but mem_mgr now hides the existence of freelists - indeed, constructing a MallocFreeAPD from a MallocFree_R_APD simply quantifies existentially over a resource vector. Our proofs first refactor the prior verification as a VSU that exports a resource-aware ASI and then use VSUConseq (and export restriction $\sqsubseteq$ from Section 3.2) to weaken the resulting VSU to a VSU that only exports a resource-ignorant ASI. We denote the latter as $\mathsf{VSU}_{\mathtt{MF}}$; the predicate $\mathsf{Mem}_{\mathsf{M}}$ gv is now revealed to be a shorthand for mem_mgr gv, parametric in a MallocFreeAPD M, and we use $\mathsf{Mem}_{\mathtt{MF}}$ gv below to refer to its instantiation for $\mathsf{VSU}_{\mathtt{MF}}$.

### 4.5 Putting it all together

Using VSULink again, we link $\mathsf{VSU}_{\mathtt{CP}}$ with a library VSU (reducing surelymalloc to malloc and the system function exit) and then with $\mathsf{VSU}_{\mathtt{MF}}$, obtaining

$$\mathsf{VSU}_{\mathtt{AppLib}} \stackrel{\text{def}}{=} \vdash^{\mathcal{S}_{\mathtt{Core}}}_{\mathsf{Mem}_{\mathtt{MF}} * N} [\,]\ \mathit{coreprog}\ [\mathcal{E}_{\mathtt{Core}}].$$

Here, *coreprog* contains all code (application plus library) with the exception of main. Note that $\mathsf{VSU}_{\mathtt{AppLib}}$'s set of imports is empty; $\mathcal{S}_{\mathtt{Core}}$ contains axiomatic specifications of OS functions such as exit and mmap.

Independent from the construction of $\mathsf{VSU}_{\mathtt{AppLib}}$ we verify main, i.e. an exemplary client or unit test, as a **semax_body** statement w.r.t. a not yet instantiated copy of $\mathcal{E}_{\mathtt{Core}}$. The specification that main is verified against a <: specialization of VST's general main_spec but is still abstract in the APDs of the application's code modules – see [14] for details.

Finally, we connect $\mathsf{VSU}_{\mathtt{AppLib}}$ with the verification of main to obtain a proof of VST's **semax_prog** statement. It is in this last proof that the satisfaction of the abstract initialization predicates for the global variables, $\mathsf{Mem}_{\mathtt{MF}}$ and $N$, is established from VST's internal initialization predicates.

## 5 Modular verification of the Subject/Observer pattern

Programs in imperative or object-oriented languages often contain callbacks: chains of function calls $A.m() \to B.n() \to A.l()$ between modules $A$ and $B$ in which $m$'s invocation of $n$ (and hence the return of control to $A$ in the call to $l$) happens when $A$'s state is *invalid*, i.e. does not satisfy $A$'s invariant. Clearly, mandating satisfaction of the invariant in $l$'s precondition – a typical requirement of API-level specifications – then prevents the verification of $n$.

A typical example is the chain update → notify → get in the subject-observer pattern, a widely used design pattern [23] that has served as a litmus test for modular specification of callback-rich programming in the literature. Figures 6 and 7 contain excerpts of a transcription of Parkinson's [55] code into[1] C. Each Subject maintains a list of subscribers – a list of observers that will be notified whenever the Subject's state is updated and then synchronize their internal state accordingly using get. The intended invariants express that each Subject's observers are in sync – a property that is violated during update's traversal of its observer list, when not-yet-notified observers are out of sync but (precisely in order to get back in sync) nevertheless invoke get.

The dominant technique for dealing with such situations in SMT-based tools employs ghost fields that track validity and unfolding of invariants and are supported by further (ghost) infrastructure that controls ownership (see e.g. [47,11]). However, this does not necessarily achieve comprehensive representation hiding: for example, the permission to violate Subject's invariant in get's precondition propagates to the precondition of notify, allowing the latter function to access the field[2] Subject.value. Furthermore, the invariant-regulating techniques typically require that SMT solving be carried out on a whole-program basis.

The flexibility of APDs to introduce multiple predicates enables an alternative in which callbacks are specified using special-purpose predicates that – similar to typestates [62] – emphasize protocol-style behavior, do not reveal the

[1] Our implementation [14] contains two further callbacks, newObs → registr → notify and registr → notify → get; the former one commences in the constructor, before any invariant has been established.

[2] For example, one may insert abstraction-violating get/putfield instructions in the subject-observer code at http://comcom.csail.mit.edu/e4pubs/{#}observer. This tool implements an advanced variant of invariance regulation using ghost instructions, semantic collaboration [59], for Eiffel. Fields are not private, and the methodology does not prevent representation exposure between such closely coupled classes.

```
/* SubjectObserver.h */
typedef struct subject *Subject;
typedef struct observer *Observer;

/* Subject.h*/
#include "SubjectObserver.h"
Subject newSubject (void);
void registr (Subject s, Observer o);
void update (Subject s, int n);
int get (Subject s);
int freeSubject(Subject s);
Observer detachfirst(Subject s);

/*Observer.h */
#include "SubjectObserver.h"
Observer newObs (Subject s);
void notify (Observer o);
int val (Observer o);
void freeObserver (Observer o);
```

```
/* Subject_rep.h */
#include "SubjectObserver.h"
typedef struct node *Node;
struct node {
  Observer obs;
  struct node * next;
};
struct subject {
  Node obs;
  unsigned value;
};

/*Observer_rep.h*/
#include "SubjectObserver.h"
struct observer {
  Subject sub;
  int cache;
};
```

**Fig. 6.** Subject/Observer: header files. The left column shows the public APIs; Subject_rep.h and Observer_rep.h are private to their respective module implementations.

```
/* Subject c */
#include "surelyMalloc.h"
#include "Observer.h"
#include "Subject.h"
#include "Subject_rep.h"

int get (Subject s) { return (s→value); };
void update (Subject s, int v) {
  s→value = v; Node n = s →obs;
  while (n) { notify(n→obs); n = n →next; } }
```

```
/* Observer.c */
#include "surelyMalloc.h"
#include "Observer.h"
#include "Subject.h"
#include "Observer_rep.h"

void notify (Observer o) {
  o →cache = get(o →sub);
  return; }
```

**Fig. 7.** Excerpts from Subject.c and Observer.c for the callback update → notify → get.

validity of module invariants, and maintain representational hiding by being just as abstract as a module's main predicate.

Concretely, our approach employs semantic subjects that are comprised of a list of observer references and a (current) value, while observers are represented as a subject pointer and the cache:

**Definition** SubjRep:= (list val) $*$ Z. **Definition** ObsRep := val $*$ Z.

Next, our APDs complement the predicates relevant for API calls by external clients, Srep and Orep, by (residual) predicates for calling the Subject functions registr, update, and get, and the Observer functions notify and val; we also introduce a predicate for the postcondition of get, GetPost:

**Record** SubjectAPD := {
Srep, RegPre, UpdPre, GetPre, GetPost: SubjRep → val → mpred;
SubjRegister: ∀ S s, Srep S s ⊢ RegPre S s;
SubjUpdate: ∀ S s, Srep S s ⊢ UpdPre S s;
SubjGetPrePost: ∀ S s, Srep S s ⊢ GetPre S s ∗ (GetPost S s -∗ Srep S s);
GetPre_ptrnull: ∀ S s, GetPre S s ⊢ !!(is_pointer_or_null s) }

**Record** ObserverAPD := { Orep, NtfPre, ValPre: ObsRep → val → mpred;
ObsNtfy: ∀ O o, Orep O o ⊢ NtfPre O o; ObsVal: ∀ O o, Orep O o ⊢ ValPre O o;
NtfPre_isptr: ∀ O o, NtfyPre O o ⊢ !!(isptr o) }

Entailment axioms such as SubjUpdate permit external clients to invoke callback functions directly but may be omitted for functions that should *only* be invoked via callbacks. The residual predicates sanction indirect invocations via callbacks without revealing the satisfaction status of module-internal invariants.

Axiom SubjGetPrePost splits Srep into a token that can (only) be used to invoke get, plus a token for reestablishing Srep from GetPost. The latter is a separating implication $-\!\!*$ rather than an entailment: it represents the requirement that an observer yields back control to its subject after completing a callback to get – the subject had retained part of its state prior to invoking notify.

To enforce these behaviors, we employ the specifications in Figures 8 and 9; again, the ASIs are parametric in all APDs mentioned, notwithstanding the mutual dependence of the modules. Using axiom SubjGetPrePost, one may show

| Function | Spec |
|---|---|
| update$(s, v)$ | $\{\mathsf{UpdPre}_{\mathsf{SP}}\ (l, z)\ s * \mathsf{Observers}\ \mathsf{NtfPre}_{\mathsf{OP}}\ s\ \mathit{vals}\ l\}$ |
| | $\rightsquigarrow \{\mathsf{Srep}_{\mathsf{SP}}\ (l, v)\ s * \mathsf{Observers}\ \mathsf{Orep}_{\mathsf{OP}}\ s\ v^{\lvert l \rvert}\ l\}$ |
| get$(s)$ | $\{\mathsf{GetPre}_{\mathsf{SP}}\ S\ s\} \rightsquigarrow \{p.\ !!(p = \mathit{snd}(S))\ \&\&\ \mathsf{GetPost}_{\mathsf{SP}}\ S\ s\}$ |

**Fig. 8.** ASI of Subject (excerpt), parametric in a SubjectAPD (SP), an ObserverAPD (OP), and a MemoryAPD (M).

that the specifications for get and notify are in subsumption relationship with large-footprint counterparts that permit invocations by external clients:

$$\{\mathsf{Srep}_{\mathsf{SP}}\ S\ s\} \rightsquigarrow \{p.\ !!(p = \mathit{snd}\ S)\ \&\&\ \mathsf{Srep}_{\mathsf{SP}}\ S\ s\}$$
$$\{\mathsf{NtfPre}_{\mathsf{OP}}\ (s, c)\ o * \mathsf{Srep}_{\mathsf{SP}}\ S\ s\} \rightsquigarrow \{\mathsf{Orep}_{\mathsf{OP}}\ (s, \mathit{snd}\ S)\ o * \mathsf{Srep}_{\mathsf{SP}}\ S\ s\}$$

The specification of update makes reference to an auxiliary Coq function that represents the "big" separating conjunction $*_{(v,o)\in \mathit{combine}(\mathit{vals},l)} P\ (s, v)\ o$,

Observers (P:ObsRep → val → mpred) (s:val) (*vals*: list Z) (l: list val): mpred.

The substantiation of these interfaces relative to our C implementations defines the main predicates as

**Definition** Srep $(l, v)\ s :=$ $\exists o.$ listrep $l\ o * s \xmapsto{\mathsf{Ews}}_{\mathsf{STP}} (o, v * \mathsf{Mtok}(\mathsf{Ews}, \mathsf{STP}, s)$.
**Definition** Orep $O\ o := o \xmapsto{\mathsf{Ews}}_{\mathsf{OTP}} O * \mathsf{Mtok}(\mathsf{Ews}, \mathsf{OTP}, o)$.

| Function | Spec |
|---|---|
| $\mathsf{newObs}(s; \mathsf{gv})$ | $\{\mathsf{RegPre}_{\mathsf{SP}}\ S\ s * \mathsf{Mem}_{\mathsf{M}}\ \mathsf{gv}\} \rightsquigarrow$ $\{p.\ \mathsf{Orep}_{\mathsf{OP}}\ (s, \mathit{snd}\ S)\ p * \mathsf{Srep}_{\mathsf{SP}}(p :: \mathit{fst}\ S, \mathit{snd}\ S)\ s * \mathsf{Mem}_{\mathsf{M}}\ \mathsf{gv}\}$ |
| $\mathsf{notify}(o)$ | $\{\mathsf{NtfPre}_{\mathsf{OP}}(s, c)\ o * \mathsf{GetPre}_{\mathsf{SP}}\ S\ s\} \rightsquigarrow$ $\{\mathsf{Orep}_{\mathsf{OP}}(s, \mathit{snd}\ S)\ o * \mathsf{GetPost}_{\mathsf{SP}}\ S\ s\}$ |
| $\mathsf{val}(o)$ | $\{\mathsf{ValPre}_{\mathsf{OP}}\ (s, c)\ o\} \rightsquigarrow \{c.\ \mathsf{Orep}_{\mathsf{OP}}\ (s, c)\ o\}$ |
| $\mathsf{freeObserver}(o; \mathsf{gv})$ | $\{\mathsf{Orep}_{\mathsf{OP}}\ O\ o * \mathsf{Mem}_{\mathsf{M}}\ \mathsf{gv}\} \rightsquigarrow \{\mathsf{Mem}_{\mathsf{M}}\ \mathsf{gv}\}$ |

**Fig. 9.** ASI of Observer, parametric in APDs SP, OP, and M.

Here, listrep is a typical list representation predicate over Node items, modeling the observers associated with a Subject. STP and OTP are shorthands for Clight's representation of the struct definitions Subject and Observer, Ews represents an exclusive writable share in VST, and Mtok(., ., .) is a variant of predicate malloc_token' from Section 4.4.

Some residual predicates are minor variants of Srep and Orep. For example,

**Definition** NtfyPre $O\ o := \mathsf{Mtok}(\mathsf{Ews}, \mathsf{OTP}, o) * \exists v.\ o \overset{\mathsf{Ews}}{\longmapsto}_{\mathsf{OTP}} (\mathit{fst}\ O, v)$.

existentially abstracts over $\mathit{snd}\ O$ but is otherwise identical to Orep. This makes validating axiom ObsNtfy trivial. As NtfyPre does not depend on a subject's value, no modification of the latter can affect the former's. Other residual predicates – like RegPre – are even definitionally equal to the main predicates, but the APD mechanism ensures that this fact is not exposed to clients.

Our C implementation permits GetPre and GetPost to actually be defined identically (indeed, getters typically don't alter data structures. . . ):

**Definition** GetPrePost $(l, v)\ s := s.\mathsf{value} \overset{\mathsf{Ews}}{\longmapsto}_{\mathsf{STP}} v * \mathsf{Mtok}(\mathsf{Ews}, \mathsf{STP}, s)$.

Here, the $p.\pi \overset{sh}{\longmapsto}_t v$ is a variant of $p \overset{sh}{\longmapsto}_t v$ that specifies the content at $p.\pi$, where path $\pi$ is a list of field names and array subscripts. Thus, GetPrePost only specifies the content of $s$.value; the remaining portion of $s$ is exactly what is retained when SubjGetPrePost splits off GetPre from a Subject. The motivation for this handling is that the invariant of the loop in update (which contains the callback to get via notify) only traverses the node list. Specifically, an invariant involving the full Srep would not ensure that the spine of the list remains unchanged, as the definition of Srep quantifies existentially over the node list. This aspect illustrates the danger of predicates that are too abstract to be useful.

Constructing VSUs for `Subject` and `Observer` proceeds straight-forwardly; we exercise VSU's support for shared libraries by first combining surelyMalloc with each of these VSUs separately, before linking the resulting VSUs with each other, with $\mathsf{VSU}_{\mathtt{MF}}$, and with a main client as described in Section 4.5.

## 5.1 Specification and proof reuse

To evaluate specification modularity and proof reuse, we verified several variations of our implementation. First, to evaluate robustness under representational change, we have Subject internally maintain a freelist of Observer nodes:

```
struct subject { Node fl; Node obs; unsigned value; };
```

The freelist is drawn upon in registr (we only invoke surely_malloc if fl is null) and replenished in detachfirst. Constructor newSubject creates an empty freelist, and freeSubject frees the entire list.

The code modification triggers new Clight ASTs, but the majority of Coq files can then simply be reprocessed: the model-level definitions, APDs, and ASIs of Subject and Observer remain unchanged, and so do the files associated with verifying Observer, linking, and main. The *only* modifications are in the implementation-dependent validation of Subject, namely in the definitions of the representation predicates and in the VST proofs of the individual functions.

Second, we verified a variant in which notify's invocation of get is replaced by a function pointer. The key code modifications are

```
/*Addition in SubjectObserver.h*/          /*Modification in Observer.h*/
typedef int (*callback)(Subject s);        void notify (Observer o, callback f);

/*Modification in Observer.c*/
void notify (Observer o, callback f) { o →cache = f(o →sub); return; };
```

The calls to notify in update and registr obtain the additional argument &get, and the specification of get can be removed from the imports of the Observer VSU. The small specification of notify becomes

$$\mathsf{notify}(o,g) : \begin{array}{l}\{\mathsf{NtfPre}\ (s,c)\ o * \mathsf{GetPre}_{\mathsf{SP}}\ S\ s * \mathsf{funcptr}'\ \phi_{\mathsf{get}}\ g\} \\ \leadsto\ \{\mathsf{Orep}_{\mathsf{OP}}(s, \mathit{snd}\ S)\ o * \mathsf{GetPost}_{\mathsf{SP}}\ S\ s\}\end{array}$$

where $\mathsf{funcptr}'\ \phi\ g$ expresses that value $g$ is a pointer to some function satisfying specification $\phi$, and $\phi_{\mathsf{get}}$ is the entry for get from Fig. 8. notify's large specification is adapted similarly. Repairing the proofs incurs changes in $< 10$ lines of Coq.

A third modification exploits VST's support for impredicative quantification to abstract over $\mathsf{GetPre}_{\mathsf{SP}}$ and $\mathsf{GetPre}_{\mathsf{SP}}$ in the definition of $\phi$, such that notify's specification is effectively parametric in suitable GetPre/GetPost pairs. Adapting the verification involves step-indexed aspects of VST and hence requires a little more work; details are included in the Coq development [14].

Finally, we verified a variation in which observers register with two subjects, as an example of a more complex interaction pattern. As this affects model-level functionality, modifications are not confined to module-internal predicate definitions but affect APDs declarations and ASI definitions. However, neither the encapsulation of representation nor the modularity of verification were compromised; supporting more than two subjects per observer would likely be similar.

## 5.2 Pattern-level specification

An alternative specification of subject-observer was proposed by Parkinson [55], who sidesteps the conflict between callbacks, modularity, and abstraction. Giving up on specifying the two classes independently, this approach defines a single abstract predicate, SubObs, that ties a subject to all its observers and yields aggregate-level function specifications. We can recover such an aggregate interface by proving that the specifications involving SubObs are abstractions (in the sense of . <: .) of the exports of the SubjectObserver VSU, generically in APDs SP and OP. Indeed, Parkinson's formulation amounts to a two-predicate APD:

**Record** AggAPD :=
{ Sub: val $\rightarrow$ list val $\rightarrow$ Z $\rightarrow$ mpred; Obs: val $\rightarrow$ val $\rightarrow$ Z $\rightarrow$ mpred }.

with specifications shown in Figure 10, using the derived notions

**Definition** SubObs $s\ O\ v := \mathsf{Sub}\ s\ O\ v * *_{o \in O} \mathsf{Obs}\ o\ s\ v$.
**Definition** $\mathsf{Obs}\_\ o\ s := \exists v.\ \mathsf{Obs}\ o\ s\ v$. *(* Obs_ is related to Obs as* $\mapsto$ _ *is to* $\mapsto$. **)*
**Definition** $\mathsf{SubObs}\_\ s\ O := \exists v.\ \mathsf{SubObs}\ s\ O\ v$.

| Function | Spec |
|---|---|
| newSubject(gv) | $\{\mathsf{Mem_M\ gv}\} \leadsto \{s.\ \mathsf{SubObs}_{\text{\_A}}\ s\ \mathsf{nil} * \mathsf{Mem_M\ gv}\}$ |
| registr($s, o$; gv) | $\{\mathsf{Sub_A}\ s\ O\ v * \mathsf{Obs}_{\text{\_A}}\ o\ s * \mathsf{Mem_M\ gv}\}$ $\leadsto \{\mathsf{Sub_A}\ s\ (o :: O)\ v * \mathsf{Obs_A}\ o\ s\ v * \mathsf{Mem_M\ gv}\}$ |
| update($s, v$) | $\{\mathsf{SubObs}_{\text{\_A}}\ s\ O\} \leadsto \{\mathsf{SubObs_A}\ s\ O\ v\}$ |
| get($s$) | $\{\mathsf{Sub_A}\ s\ O\ v\} \leadsto \{v.\ \mathsf{Sub_A}\ s\ O\ v\}$ |
| newObs($s$; gv) | $\{\mathsf{SubObs_A}\ s\ O\ v * \mathsf{Mem_M\ gv}\} \leadsto \{p.\ \mathsf{SubObs_A}\ s\ (p :: O)\ v * \mathsf{Mem_M\ gv}\}$ |
| notify($o$) | $\{\mathsf{Sub_A}\ s\ O\ v * \mathsf{Obs}_{\text{\_A}}\ o\ s\} \leadsto \{\mathsf{Sub_A}\ s\ O\ v * \mathsf{Obs_A}\ o\ s\ v\}$ |
| val($o$) | $\{\mathsf{SubObs_A}\ s\ O\ v\} \leadsto \{v.\ \mathsf{SubObs_A}\ s\ O\ v\}$ |

**Fig. 10.** Selected aggregate specifications, parametric in an AggAPD A. Except for the occurrence of $\mathsf{Mem_M}$ gv, the specifications coincide with Parkinson [55]'s specifications.

Constructing an AggAPD A from a SP/OP pair is trivial: take Sub to be $\mathsf{Srep_{SP}}$ and Obs to be $\mathsf{Orep_{OP}}$; proving the . <: . lemmas is then straight-forward.

SubObs constitutes a *pattern invariant*, or the pattern's primary predicate, with residuals Sub and Obs. From the aggregate's point of view, update $\rightarrow$ notify $\rightarrow$ get is not a callback but an internal nesting of invocations, so the small-footprint specifications typically don't pose a problem for existing methodologies; client-visible specifications with large footprints can be derived using the frame rule. In this sense, the pattern reestablishes "sequential atomicity" of operations. Exploring whether other design patterns can be similarly derived from the ASIs of their constituent classes is a topic for future research: *are typical design patterns the abstraction units at which sequential atomicity is reestablished, callbacks at most occur in valid states, and residual predicates are avoided?*

An aggregate specification for the function pointer implementation from Section 5.1 can be obtained using a modified AggAPD, with residual predicates GetPre etc.. But a better option is to remove the pattern-internal functions notify, registr, and perhaps even get from the aggregate ASI. In fact, notify's new signature reveals the use of function pointers, hence even an aggregate-level specification would have to include $\mathsf{funcptr'}\ \phi\ g$ terms. Thus, we instead employ the notion $\sqsubseteq$ from Section 3.2 to lift the VSU for SubjectObserver with function pointers from Section 5.1 to a VSU for the aggregate but narrowed ASI and then reverify main w.r.t the latter.

## 6 Verification of object principles

This section considers features that – together with state encapsulation and modularity – are cornerstones of object orientation: the ability for (instances of) multiple implementations of an interface to dynamically coexist and interact, dynamic dispatch, subtyping, *self*, and inheritance. To maintain the dataless discipline, we employ a uniform but simple object encoding that is typical for industrial and open-source C developments: dynamic dispatch is implemented using function pointers that are bundled into separate **struct**s (method tables) that are accessible as the first element of the object representations. Subtyping – providing additional methods – and representation inheritance are modeled by extending these **struct**s, respectively, but are orthogonal to each other, and only the former one is exposed in APIs. In the second half of this section, we hide the dynamic dispatch mechanism behind a wrapper interface. We specify objects by reference to a semantic (Coq-level) object model, thus comprehensively separating object reasoning from C-level reasoning: constructors establish, and methods maintain, abstract object predicates that clients need not (and cannot) unfold.

We again proceed in stages, using the widely used running example of points located on a one-dimensional axis (see e.g. [18]). Figure 11 shows a preliminary API for basic, bumpable, and colored points, organized in a simple subtyping relationship. We provide multiple implementations for each interface (using different data representations), each exposing its set of constructors in a separate header file - Figure 12 shows implementation **I1**. Clients select an implementation during object creation but cannot otherwise distinguish between them: method dispatch selects the appropriate function from the method table, as in

```
typedef struct point *Point;
struct methods {
  int (*get) (Point);
  void (*set) (Point, int); };
typedef struct methods * Methods; typedef struct bpoint *BPoint;
struct bmethods {
  int (*get) (Point);
  void (*set) (Point, int);
  void (*bump) (BPoint); };
typedef struct bmethods * BMethods;
```

```
typedef struct bmethods * BMethods; typedef struct cpoint *CPoint;
struct cmethods {
  int (*get) (Point);
  void (*set) (Point, int);
  void (*bump) (BPoint);
  int (*getC) (CPoint); };
typedef struct cmethods * CMethods;
struct point { Methods mtable; };
struct bpoint { BMethods mtable; };
struct cpoint { CMethods mtable; };
```

**Fig. 11.** PointInterface.h, containing three interfaces for one-dimensional points

```
BPoint bp = makeBPoint_I1(4); int i = ((BMethods)(bp→mtable))→get((Point)bp)).
```

```
struct point_I1 {              struct bpoint_I1 {           struct cpoint_I1 {
  Methods mtable;                BMethods mtable;             CMethods mtable;
  int value; };                  int value; };                int value; int color; }; int get_I1 (Point p) { return (((struct point_I1 *)p)→value); }
void set_I1 (Point p, int i) { ((struct point_I1 *)p)→value = i; return; }
void bump_I1 (BPoint p) { ((struct bpoint_I1 *)p)→value++; return; }
int getC_I1 (CPoint p) { return (((struct cpoint_I1 *)p)→color); }
BPoint makeBPoint_I1 (int i) {
  struct bpoint_I1 *p = (struct bpoint_I1 *)surely_malloc(sizeof *p);
  BMethods m = (BMethods)surely_malloc(sizeof *m);
  m →get = &get_I1; m →set = &set_I1; m →bump = &bump_I1;
  p →value = i; p →mtable = m; return ((BPoint)p); }
```

**Fig. 12.** Implementation **I1**. Constructors makePoint_I1 and makeCPoint_I1 omitted. A second implementation **I2** employs representations point_I2 etc. and exposes constructors makeBPoint_I2 etc.

The basis of object specifications is a general method table predicate:

$$\mathsf{MTable}(T, k, names, m, specs, \mathcal{I}) \mathrel{\widehat{=}} \mathsf{Mtok}(\mathsf{Ews}, k, m) * \\ \exists\, \pi.!!(\mathsf{readable}(\pi))\ \&\&\ *_{(\mu,\phi)\in names \times specs}\ \exists\, v.\mathsf{funcptr}(\phi\mathcal{I}, v) * m.\mu \mapsto^{\pi}_{k} v.$$

It asserts that the **struct** $m$ (of shape $k$) contains at field names *names* pointers to functions satisfying *specs*, where $\mathcal{I}$ is of Coq-type $\mathsf{Pred(T)} \mathrel{\widehat{=}} (\mathsf{T}{*}\mathsf{val}) \to \mathsf{mpred}$ and *specs* has type $\mathsf{list\ (Pred(T)} \to \mathsf{funspec)}$. A generic object layout predicate

$$\mathcal{N}\ T\ tbl\ (\sigma\ k : \mathsf{type})\ names\ specs\ (x : T * val) : \mathsf{mpred}\ = \\ \exists\, \delta\, \mathcal{I}.\ \mathcal{I}\ x * \mathsf{Mtok}(\mathsf{Ews}, \delta, snd\ x) * \\ \exists\, m.\, (snd\ x).tbl \mapsto^{\mathsf{Ews}}_{\sigma} m\ *\ \mathsf{MTable}(T, k, names, m, specs, \mathcal{I})$$

then combines a specified method table (located at field *tbl*) with the requirement that the (memory identified by the) object pointer satisfy $\mathcal{I}$. C types $\sigma$ and $\delta$ represent the object's static and dynamic types. The joint use of $\mathcal{I}$ in MTable and $\mathcal{N}$ ensures that an object's methods agree with its data component on what representation predicate should be maintained. The existential abstraction over $\mathcal{I}$ ensures representation hiding: external clients merely see a invariant of (Coq) type $T$. Thus, different C implementations of an object interface may employ different representations but still satisfy the same external specification.

Specifically, we introduce Coq-level object interface types in the style of Hofmann and Pierce's object model [30]:

```
Record PointM (X:Type):Type := { get : X → Z; set : X → Z → X; }
Record BPointM (X:Type):Type :=
  { PointM_of_BPointM :> PointM X; bump : X → X; bumpable : X → Prop; }.
Inductive Color:Type := blue | red | green.
Record CPointM (X:Type):Type :=
  { BPointM_of_CPointM :> BPointM X; getC: X → Color; color_code: Color → Z; }.
```

The parameters $X$ represent *semantic* object representations. On the one hand, we may instantiate these and define Coq-level behaviors, like m1, bm1, cm1:

**Record** PointRep := { value : Z }.
**Record** CPointRep := { pointRep :> PointRep; color : Color }.
**Definition** m1: PointM PointRep :=
{| get := fun s ⇒ value s; set := fun s i ⇒ {| value := i |} |}.
**Definition** bm1: BPointM PointRep :=
{| PointM_**of**_BPointM := m1; bump := fun s ⇒ {| value := value s + 1 |};
bumpable := fun s ⇒ min_signed ≤ value s < max_signed |}.
**Definition** cm1: CPointM CPointRep := {| ... *(∗details omitted∗)* |}

But the interface types also enable specifications for $\mathsf{get}(p)$ and $\mathsf{set}(p, j)$:

$$\mathsf{get\_spec}\ T\ (P : \mathsf{PointM}\ T) \mathrel{\hat{=}} \lambda \mathcal{I} : \mathsf{Pred}\, T.\ \{\mathcal{I}(t,p)\} \leadsto \{\mathsf{get}\ T\ P\ t.\ \mathcal{I}(t,p)\}$$
$$\mathsf{set\_spec}\ T\ (P : \mathsf{PointM}\ T) \mathrel{\hat{=}} \lambda \mathcal{I} : \mathsf{Pred}\, T.$$
$$\{\mathsf{min\_signed} \leq j \leq \mathsf{max\_signed}\ \&\ \mathcal{I}(t,p)\} \leadsto \{\mathcal{I}(\mathsf{set}\ T\ P\ t\ j, p)\}$$

Thus, each method has a Coq-level counterpart that is parametric in (semantic) representations and behaviors. To specify the constructors, we first define specializations of $\mathcal{N}$ for the three interfaces by instantiating with the appropriate method specifications and syntactic elements:

$$\mathcal{P}\ T\ (P : \mathsf{PointM}\ T) : \mathsf{Pred}\, T \mathrel{\hat{=}}$$
$$\mathcal{N}\ T\ \mathtt{mtable}\ \mathtt{point}\ \mathtt{methods}\ [\mathtt{get}; \mathtt{set}]\ [\mathsf{get\_spec}\ T\ P; \mathsf{set\_spec}\ T\ P]$$
$$\mathcal{B}\ T\ (B : \mathsf{BPointM}\ T) : \mathsf{Pred}\, T \mathrel{\hat{=}}$$
$$\mathcal{N}\ T\ \mathtt{mtable}\ \mathtt{bpoint}\ \mathtt{bmethods}\ [\mathtt{get}; \mathtt{set}; \mathtt{bump}]$$
$$[\mathsf{get\_spec}\ T\ B; \mathsf{set\_spec}\ T\ B; \mathsf{bump\_spec}\ T\ B]$$
$$\mathcal{C}\ T\ (C : \mathsf{CPointM}\ T) : \mathsf{Pred}\, T \mathrel{\hat{=}}$$
$$\mathcal{N}\ T\ \mathtt{mtable}\ \mathtt{cpoint}\ \mathtt{cmethods}\ [\mathtt{get}; \mathtt{set}; \mathtt{bump}; \mathtt{getC}]$$
$$[\mathsf{get\_spec}\ T\ C; \mathsf{set\_spec}\ T\ C; \mathsf{bump\_spec}\ T\ C; \mathsf{getC\_spec}\ T\ C]$$

Here, `point`, `bpoint`, `cpoint` and `methods`, `bmethods`, `cmethods` are the **struct**s defined in the header file (Figure 11) and `mtable`, `get`, . . . , `getC` are the field names in these **struct**s. The exemplary spec for base point constructors is then

$$\mathsf{makePoint}(i; \mathsf{gv}) : \{\mathsf{min\_signed} \leq i \leq \mathsf{max\_signed}\ \&\ \mathsf{Mem}_{\mathsf{M}}\ \mathsf{gv}\}$$
$$\leadsto \{p.\ \mathsf{Mem}_{\mathsf{M}}\ \mathsf{gv} * \mathcal{P}\ T\ P\ (\mathsf{Init\_Point}(i), p)\}.$$

Verifying **I1** and **I2** then yields VSUs whose export interfaces tie makePoint_I1 makePoint_I2 to the specialization of this constructor to $P :=$ m1, and similarly for the other constructors. The resulting objects behave indistinguishably; the existential quantification over $\mathcal{I}$ in the definition of $\mathcal{N}$ carries over to $\mathcal{P}$, $\mathcal{B}$, and $\mathcal{C}$, ensuring that the representational differences between **I1** and **I2** are hidden from clients: when verifying a method call, clients unroll $\mathcal{P}$ etc., but each time receive a "fresh" symbolic representation predicate $\mathcal{I}$.

*Wrapper-based verification* The unrolling of object predicates corresponds to the exposure of the method table in our API. Programmatically, better encapsulation is provided by wrappers that hide the function pointer mechanism, like

**int** GET (Point p) { Methods m = p→mtable; **return** (m→get(p)); }

The header file for these wrappers resembles the API of an ADT, but merely disguises object-orientation: we still support multiple implementations (using the same constructors as above), and operations are still invoked using dynamic dispatch. On the specification side, wrappers can be modeled as an APD

**Record** WrapperAPD := { Wr_Pt: ∀ T, PointM T → Pred T;
Wr_BPt: ∀ T, BPointM T → Pred T; Wr_CPt: ∀ T, CPointM T → Pred T }.

with one constructor per interface, in resemblance to the use of class names to index predicate families [56]. The VSU for the wrapper then encapsulates the object predicates $\mathcal{P}$ etc., exporting an ASI with specifications such as

$$\mathsf{GET}(p) : \begin{array}{l} \{\mathsf{Wr\_Pt}\ W\ T\ P\ (t,p)\} \leadsto \{\mathsf{get}\ T\ P\ t.\ \mathsf{Wr\_Pt}\ W\ T\ P\ (t,p)\} \wedge \\ \{\mathsf{Wr\_BPt}\ W\ T\ P\ (t,p)\} \leadsto \{\mathsf{get}\ T\ P\ t.\ \mathsf{Wr\_BPt}\ W\ T\ P\ (t,p)\} \wedge \\ \{\mathsf{Wr\_CPt}\ W\ T\ P\ (t,p)\} \leadsto \{\mathsf{get}\ T\ P\ t.\ \mathsf{Wr\_CPt}\ W\ T\ P\ (t,p)\} \end{array}$$

We can further improve client-side usability by replacing these intersection specifications by a deep embedding of the three interface alternatives; this eliminates a corresponding case distinction in client-side proofs, when symbolic execution reaches the invocation of a wrapper function. As an example, we verified a linked list module that permits insertion of basic, bumpable, or colored points and provides map operations that apply $\mathsf{SET}$, $\mathsf{BUMP}$, ... to all elements. Each element may internally employ **I1** or **I2**. Of course, the precondition of mapping $\mathsf{BUMP}$ requires all elements to be of dynamic type (at least) $\mathsf{BPoint}$ and have a bumpable coordinate; however, this condition emerges as a constraint on semantic objects and can be discharged without unfolding object representation predicates.

*Self and late binding* Verification using the above constructions fails for methods whose body contains virtual calls on *self*: the definition of $\mathcal{N}$ effectively separates the object's data region from the method table upon method entry, making only the former accessible inside the body. To overcome this limitation, we define a variant of $\mathcal{N}$ using the higher-order recursive functor

$$\begin{array}{l} \mathcal{F}\ (\mathcal{I}\ X : \mathsf{Pred}\,T) : \mathsf{Pred}\,T \mathrel{\widehat{=}} \\ \quad \lambda(x : T * val).\ \exists\, \delta\, m.\ \mathcal{I}\ x * \mathsf{Mtok}(\mathsf{Ews}, \delta, snd\ x) * (snd\ x).tbl \mapsto_{\sigma}^{\mathsf{Ews}} m \\ \qquad\qquad * \rhd \mathsf{MTable}(T, k, names, m, specs, X) \end{array}$$

in which $\mathcal{I}$ is now a parameter (we eschew the parameters $T, \ldots, specs$ for readability) and $X$ plays the role of $\mathcal{N}$. Recursion via $X$ is protected by VST's [4] modality $\rhd$; indeed, any access to a method table inside a method happens at least one step *later* than the method's own invocation. Contractiveness of $\mathcal{F}$ (proven in VST) ensures the existence of a fixed point $\widehat{\mathcal{F}}(\mathcal{I}) := HORec(\mathcal{F}(\mathcal{I}))$. Recovering the quantification over $\mathcal{I}$, we then replace $\mathcal{N}$ with $\mathcal{N}^* := \exists \mathcal{I}.\ \widehat{\mathcal{F}}(\mathcal{I})$. With this modification in place, one may verify virtual calls on *self*, like a variant of **I1** that implements bump using get and set (still w.r.t. m1, bm1, and cm1).

An important application of *self* is (observably behavior-altering) method overriding. At the semantic level, Hofmann and Pierce explicate how positive subtyping supports both early and late binding variants of overriding; these differ in whether the observable behavior of bump (when implemented in terms of get and set) is affected when a subclass subsequently overrides set to, say, reset the coordinate to 0. Furthermore, method overriding may affect how functions defined in a superclass act on subclass-introduced state components. For example, one may impose that updating the coordinate turns a point's color blue. Semantically, all these variations yield novel behaviors m2, bm2, and cm2, etc. that can be compared to the earlier behaviors using Hofmann and Pierce's theory. As a consequence of our two-level reasoning, and the choice to parameterise constructor/method specifications by behaviors, we can leverage their techniques: implementations **I3**, **I4**... that realize the overriding variants can be verified as further VSUs for our earlier export interface, by (now) specializing the constructor specifications to m2, etc.. Afterwards, the modified behaviors propagate through dynamic dispatch and wrappers as expected, permitting clients of e.g. the list module to map bump over elements with different behavior. Side conditions during symbolic method calls refer exclusively to semantic objects and behaviors, do not necessitate the unrolling of representation predicates, and can often just be discharged using simplification.

## 7 Discussion

*Related and future work* Certified Abstraction Layers (CAL, [24,26]) are used in the CertiKOS project [25] to verify feature-rich operating system kernels and hypervisors in Coq. CAL permits horizontal and vertical composition of components, and establishes full abstraction between the imports and exports. CAL's methodology was recently rephrased as a synthesis from a systems-oriented DSL, DeepSEA, to C, with a CompCert backend [64]. However, "(T)here is no use of C pointers and no built-in support of dynamic memory allocation (every DeepSEA object is realized as a set of static variables), so programs that need dynamic allocation will have to implement it themselves" ([64], page 10). While this fragment remarkably suffices for the intended application area, it is unlikely to satisfy general-purpose programmers or compiler writers for other systems languages.

Ironclad Apps and Ironfleet [29,28] are systems based on Dafny and TLA+ for verifying safety and liveness of distributed systems, and app security. By connecting model-level, concurrency-aware reasoning, state-machine refinement, and Floyd-Hoare verification, their approach provides abstraction-bridging functionality similar to that of proof-assistant-based reasoning, trading off TCB size and foundational integration in an logical framework against automation and developer productivity. Ironclad Apps compile to verified assembly; Ironfleet employs a formally unverified route via Dafny and the .NET compiler for C#.

Überspark [67] is a system based on Frama-C and SMT for compositionally verifying commodity system software written in C and assembly. Überspark's primary applications are hypervisor components and OS kernels, but it currently addresses only safety and security properties (memory separation, control-flow integrity, information flow) rather than functional correctness. The same limitation applies to proof-carrying code systems [49,3,6,13,27], at (virtual) machine or assembly level. Several PCC systems proposed hierarchies of formalisms that connect operational semantics, a general-purpose program logic, and tactical checkers or algorithmic inference systems for higher-level type systems, abstract interpretation, or program analyses [16,2,17,1]. VST's tactical automation is optimized for symbolic execution and functional correctness, but the underlying proof rules could equally well be used to prove soundness of static analyses or code synthesizers; we expect our structuring principles for separate compilation will be just as useful in these scenarios as they are for functional correctness.

McKinna and Burstall [45] pioneered the use of existential abstraction to formally tie programs to their specifications and proofs in a modern proof assistant. VSU realizes aspects of their vision of deliverables for a mainstream language but is at this point not endowed with similarly rigorous categorical underpinnings.

Representation hiding in separation logic can also be obtained using hypothetical frame rules [54,10], but no such rule is provided by VST at present. Pragmatically, the two approaches appear complementary: modules that expose interesting state (e.g. a list ADT, the point objects,...) favor existential abstraction/APDs, as clients can access associated reasoning principles on demand, at specific program points. In contrast, modules like the resource-unaware memory manager might benefit from hypothetical framing: the predicate $\mathsf{Mem}_{\mathsf{M}}\ \mathsf{gv}$ carries no client-relevant information but still needs to be carried around in many function specifications in our treatment.

VST's specification subsumption resembles behavioral subtyping [44,42], a notion commonly used in verification tools for Java-like languages for relating specifications across a class hierarchy. Exploring the relationship between our use of positive subtyping, other notions of subtyping and inheritance, and Liskov's Substitution Principle [43] constitutes future work.

By supporting field update, Hofmann and Pierce's theory addresses shortcomings of purely functional object models, but its support for object aggregates or complex ownership structures appears limited and not much studied. A two-level encoding could likely also be developed for concurrency-inspired object models [33,32,31], perhaps by adapting the theory of interaction trees [68,39]. However, VST's partial-correctness interpretation of triples limits the end-to-end usefulness of coinductive reasoning. A recent proposal for integrating statically typed Smalltalk-inspired objects into a functional calculus is Wyvern [52].

In the context of SMT-based verification tools, Parkinson and Bierman [57] highlight examples that go beyond behavioral subtyping, and Summers et al. [66] identify a catalog of advanced uses of class invariants. We intend to apply VST to the former soon; a better understanding of the latter could perhaps commence by recasting Drossopoulou et al.'s general framework for object invariants [22] in separation logic. However, some aspects of class/object invariants may not immediately transfer from Java-like to Smalltalk-style object models.

In Java, an object's representation remains constant over its lifetime. By separately quantifying over $\mathcal{I}$, our pre- and postconditions may support dynamic representation change a la Fickle [21] (with suitable updates to the method table), as long as both representations fit into an object's top-level **struct**.

Krishnaswami et al. [41] verify subject-observer and other patterns (iterators, flyweight, factory) by equipping a functional language, Idealized ML, with effectful specifications based on higher-order separation logic. Their verification was partially formalized in a predicative Hoare Type Theory/Ynot and employs abstract module definitions that combine code and specification. Their use of separating implication can likely be transferred to our setting, but their implementation does not separate the functionality of subjects and observers to same extent and thus does not raise the same specification challenges. Considerate reasoning [65], object propositions [51], and multi-object languages such as Rumer [9] are alternatives in the design space spanned by invariant techniques, aliasing/separation and ownership; all validate variants of Composite pattern.

Extrapolating from our exploration of the Composite pattern, it appears feasible to generate VST specifications, loop invariants, and APD declarations from Verifast [34]; synthesizing full proofs will be more challenging.

Object encodings in the Linux kernel, GTK/GObject, or the SQLite database engine deviate from the Smalltalk tradition and expose APIs that are not fully dataless. We suspect these systems also differ from standard language-level object disciplines in their need for deeply layered ownership control or model-level object aggregates. Like Schreiner's encoding [63], these systems thus provide interesting opportunities for future case studies.

*Conclusion* The ability of type theory to capture modularity and abstraction is well-established. But while, e.g. Mitchell and Plotkin's insight has been highly influential in the world of functional programming, it has not yet made its way into verification tools for mainstream languages. Taking inspiration from their work, we introduced Verified Software Units as a general component calculus for VST, and developed an infrastructure for separating the declarations of abstract predicates from concrete predicate definitions. We showed that residual predicates support callbacks which violate operation atomicity, as is the case in the subject-observer pattern. Finally, we introduced a two-level approach to specifying object principles, yielding a simple logic for Smalltalk-style objects in C. Together, these innovations substantially advance VST's capability to verify modular C developments that employ diverse programming styles.

*Acknowledgments:* This work was funded by the National Science Foundation under the awards 1005849 (*Verified High Performance Data Structure Implementations*, Beringer) and 1521602 *Expedition in Computing: The Science of Deep Specification*, Appel). The author is grateful to the members of both projects for their feedback and greatly appreciates the reviewers' comments and suggestions.
